# Nodal involvement in patients with small, clinically node-negative HER2-positive breast cancer after staging with FDG-PET/CT and neoadjuvant systemic therapy

**DOI:** 10.1016/j.breast.2024.103822

**Published:** 2024-10-18

**Authors:** Josefien P. van Olmen, Veerle CM. Geurts, Marie-Jeanne TFD. Vrancken Peeters, Caroline A. Drukker, Marcel PM. Stokkel, Marleen Kok, Frederieke H. van Duijnhoven

**Affiliations:** a Department of Surgical Oncology, Netherlands Cancer Institute / Antoni van Leeuwenhoek, Amsterdam, the Netherlands; b Department of Tumor Biology & Immunology, Netherlands Cancer Institute / Antoni van Leeuwenhoek, Amsterdam, the Netherlands; c Department of Surgery, Amsterdam University Medical Center, Amsterdam, the Netherlands; d Department of Nuclear Medicine, Netherlands Cancer Institute / Antoni van Leeuwenhoek, Amsterdam, the Netherlands; e Department of Medical Oncology, Netherlands Cancer Institute / Antoni van Leeuwenhoek, Amsterdam, the Netherlands

**Keywords:** Breast cancer, FDG-PET/CT, Neoadjuvant treatment, HER2 positive

## Abstract

**Background:**

Guidelines recommend systemic therapy for stage I HER2+ breast cancer (BC). Neoadjuvant systemic treatment (NAST) allows response-guided adjuvant treatment. However, prior to NAST only clinical nodal staging is available, risking undertreatment if ypN+ is observed. Here, we aim to evaluate the impact of FDG-PET/CT and NAST on nodal disease status in patients with small, node-negative HER2+ BC.

**Methods:**

This retrospective study included patients with small (≤3 cm), clinically node-negative HER2+ BC diagnosed between 2011 and 2023. Primary outcome was the proportion of patients with nodal disease on final pathology after upfront surgery or NAST followed by surgery with or without FDG-PET/CT. Patients received either paclitaxel + trastuzumab (PT) or a more extensive regimen.

**Results:**

Of the 370 included patients, 183 underwent FDG-PET/CT, detecting regional or distant metastases in 14 patients (7.7 %).

Among 356 patients with cN0 disease, 44.1 % (n = 157/356) had upfront surgery, with only 3 % (5/157) having an FDG-PET/CT. The remaining 55.9 % (199/356) started with NAST, with 82 % (n = 164/199) having an FDG-PET/CT. Among patients treated with NAST, 36 % received PT.

Nodal involvement on pathology was seen in 19.1 % (n = 29/152) after upfront surgery without FDG-PET/CT and 6.1 % (10/164) after NAST combined with FDG-PET/CT.

After NAST, 58 % had a pCR (PT: 49 %, other: 63 %). Nodal involvement on final pathology was seen in 6.9 % after PT and in 5.5 % after more extensive regimen.

**Conclusions:**

The proportion of patients with ypN + after NAST combined with FDG-PET/CT was only 6.1 %. Neoadjuvant treatment can be a safe treatment strategy for patients with stage I HER2+ BC.

## Highlights

• Population: small (≤3 cm), clinically node negative, HER2-positive breast cancer.
• Nodal involvement after neoadjuvant systemic treatment and FDG-PET/CT was 6.1 %.
• Nodal involvement after upfront surgery without FDG-PET/CT was 19.1 %.

## Abbreviations

ALND = Axillay lymph node disection BC =: Breast cancer
ER =: Estrogen receptor
FDG-PET/CT =: Fluorodeoxyglucose-positron emission tomography/computed tomography
FNA =: Fine needle aspiration
iDFS =: Invasive disease free survival
ITC =: Isolated tumor cells
NAST =: Neoadjuvant systemic therapy
OR =: Odds ratio
pCR =: Pathological complete response
rCR =: Radiological complete response
PT =: Paclitaxel + trastuzumab
RT =: Radiotherapy
TDM-1 =: Trastuzumab emtansine

## 1 Introduction

The management of small node-negative HER2-positive (HER2+) breast cancer (BC) traditionally consists of upfront surgery followed by adjuvant systemic therapy with or without radiotherapy (RT) [1]. Until 2015, no standard treatment regimen was recommended for patients with stage I HER2+ BC. However, Tolaney et al. showed a 3-year invasive disease free survival (iDFS) of 98.7 % with single-agent adjuvant paclitaxel and trastuzumab (PT) for patients with T ≤ 3 cm N0 disease [2]. Long-term follow-up of 406 patients revealed 10-years iDFS of 91.3 % and 10-years breast cancer specific survival of 98.8 % [3]. Based on these results, adjuvant PT is currently the recommended treatment regime for stage I HER2+ BC [4].

In contrast to stage I, patients with stage II or III HER2+ BC are preferably treated with neoadjuvant systemic treatment (NAST) allowing response-guided treatment in the adjuvant setting [5]. As investigated in the Katherine trial and the ATEMPT trial, adjuvant trastuzumab emtansine (T-DM1) was associated with improved iDFS in case of residual disease after NAST [[6], [7], [8], [9]].

However, a dilemma can occur when treating clinically stage I patients with PT in the neoadjuvant setting. Although promising results with neoadjuvant PT were observed, as 32.9 % of patients reached pathological complete response (pCR) [10], the lack of pathological nodal status can result in undertreatment since patients with pN + would be offered combination chemotherapy and dual HER2-blockade. A notable proportion of patients stage 1 HER + BC who undergo upfront surgery has lymph node involvement, ranging from 17% to 23 % [[10], [11], [12]]. In addition, data are lacking with respect to the exact benefit of adjuvant strategies in case of non-pCR after neoadjuvant PT.

In recent years, FDG-PET/CT has gained a more prominent role in staging BC, especially for its high specificity and sensitivity in the detection of axillary and regional lymph node metastasis. Although FDG-PET/CT is recommended for all clinical stage IIB-III BC patients, little is known about the benefit of FDG-PET/CT in patients with stage I HER2+ node-negative BC [[13], [14], [15]].

We hypothesized that the addition of FDG-PET/CT to the diagnostic work-up for patients with small, HER2+ BC who were lymph node-negative on ultrasound, could improve clinical staging, leading to less nodal involvement on final pathology and better patient selection for a less intense NAST treatment schedule including PT. In this study, we investigated clinical upstaging by FDG-PET/CT compared to ultrasound alone and nodal involvement on final pathology in patients treated with NAST with or without FDG-PET/CT for clinical staging. We compared these nodal involvement rates with those in patients treated with upfront surgery.

## 2 Methods

### 2.1 Patient selection

In this retrospective cohort study, patients diagnosed with small (≤3 cm), clinically lymph node-negative (cN0) HER2+ BC (defined as IHC 2+ with ISH amplification or IHC 3+), between January 2011 and September 2023 in the Netherlands Cancer Institute were included. The included patients were treated with either upfront surgery or NAST. Tumor size was determined using MRI or ultrasound. All patients were cN0 at time of patient selection based on ultrasound. In case of suspected lymph nodes on ultrasound, but representative negative fine needle aspiration (FNA) or biopsy, a patient was considered to be cN0. We included both patients with and without FDG-PET/CT-scan included in their diagnostic work-up, which was performed after ultrasound confirmed cN0-status and before start of treatment (NAST or upfront surgery). Patients were excluded if distant metastases were already suspected (M1), if they had a history of ipsilateral DCIS or BC, a bilateral HER2+ tumor, multiple ipsilateral tumors of different subtype, no nodal surgery, recurrent BC, and/or a HER2-tumor upon revision.

## 3 Outcomes

The primary outcome was the effect of staging with FDG-PET/CT in patients treated with NAST defined as:

• The proportion of patients who were cN0 on ultrasound and upstaged to cN + or cM + by FDG-PET/CT.
• Nodal involvement on final pathology after NAST in patients who were cN0 on both ultrasound and FDG-PET/CT compared to ultrasound only.

Secondary outcomes were:

• Nodal involvement on final pathology after upfront surgery in cN0 patients.
• Nodal involvement on final pathology and pCR-rates in cN0 patients treated with neoadjuvant PT compared to a more intense neoadjuvant regimen.
• Factors associated with nodal involvement on final pathology

### 3.1 Staging by FDG-PET/CT

According to our local guidelines, an FDG-PET/CT-scan is recommended for all breast cancer patients who have an indication for neoadjuvant systemic therapy, including patients with small breast tumors (<3 cm). Some patients did not undergo an FDG-PET/CT for clinical staging due to physician's decision or because they were referred from another center. Approximately 1 h after the administration of 18F-FDG, PET/CT scanning (Philips Gemini TF Big Bore, Cleveland, OH, USA) was performed from the base of the skull to the groin region. If a FDG-PET/CT was performed, reports were reviewed to determine the presence of FDG-PET/CT-positive lymph nodes and/or distant lesions and whether the presence of positive LN and/or distant lesion resulted in clinical upstaging after FDG-PET/CT scan. For the patients who were clinically upstaged after FDG-PET/CT, additional information about change of local and/or systemic treatment due to upstaging was obtained.

### 3.2 Data collection

For all patients, electronic patient files were reviewed to obtain patient- and, tumor characteristics, as well as treatment-related data such as local therapy (surgery, radiotherapy) and systemic treatment (anti-HER2 therapy, chemotherapy, hormonal therapy). Estrogen-positivity (ER+) was defined as ≥10 % ER expression. Additionally, pathology reports were reviewed to assess pathological breast- and nodal disease after primary treatment. If patients received NAST, breast disease status was classified as pCR or non-pCR. Patients with residual DCIS without invasive component after NAST were considered having a pCR. Nodal pathology was classified as either (y)pN0 (including the presence of isolated tumor cells) or (y)pN+ (including micro and macro metastases) [16].

### 3.3 Statistical analysis

Descriptive analyses were performed on categorical data (frequency and percentages) and continuous data (median and interquartile range). Group comparisons were performed using Mann-Whitney *U* test for continuous data and the Chi-square test for categorical data. A logistic regression method was used to evaluate the relationship between characteristics and nodal involvement on final pathology and pathological complete response, using univariable and multivariable analysis. Variables were included in the multivariable analysis if they had a p-value less than 0.100 in the univariable analysis or were considered clinically relevant. All statistical analyses were conducted using SPSS, version 27 (IBM Corp., Armonk, N.Y., USA).

## 5 Results

### 5.1 Cohort description

**Fig. 1:**
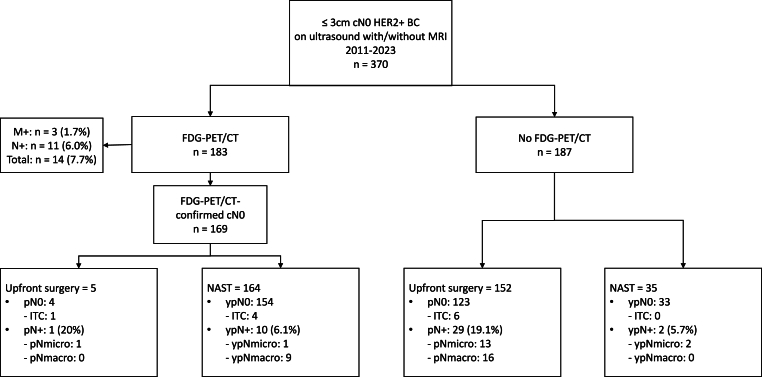
Flowchart of nodal involvement in all patients with small (≤3 cm), clinically node-negative on ultrasound, HER2-positive breast cancer after neoadjuvant systemic treatment or upfront surgery with or without staging by FDG-PET/CT. BC, breast cancer; cN0, clinically node-negative; M+, distant metastases on FDG-PET/CT; N+, regional metastases on FDG-PET/CT; NAST, neoadjuvant systemic treatment; pN0, pathological node-negative after primary surgery; pN+, pathological node-positive after primary surgery; ypN0, pathological node-negative after neoadjuvant systemic treatment; ypN+, pathological node-positive after neoadjuvant systemic treatment.

We identified 370 patients with small (T ≤ 3 cm, N0) HER2+ BC diagnosed between January 2011 and September 2023 who met eligibility criteria (Fig. 1). Median age at diagnosis was 50 years (IQR: 41.75–59), most patients had a unifocal tumor (78.9 %) of non-special histological subtype (91.9 %) and most tumors were grade 3 (51.4 %) and ER+ (74.1 %). (Table 1). All patients underwent ultrasound of the axilla on which 32 % had suspected axillary lymph nodes, for which FNA or biopsy was performed, resulting in negative pathology. The majority (87 %) underwent MRI prior to treatment.

**Table 1:** Patient-, tumor and treatment characteristics of all included patients. n = 370.

	Total N = 370	NAST N = 213	Upfront surgery N = 157	p-value
**Median age (IQR)**	50 (41.75–59)	48 (39.5–57)	53 (44–62)	**<0.001**^**1**^
**Tumor grade**				0.299^2^
1	11 (3.0)	6 (2.8)	5 (3.2)	
2	163 (44.1)	90 (42.3)	73 (46.5)	
3	190 (51.4)	111 (52.1)	79 (50.3)	
Missing	6 (1.7)	6 (2.8)	0	
**cT status**				**<0.001**^**2**^
1a	9 (2.4)	2 (1.0)	7 (4.5)	
1b	38 (10.3)	8 (3.8)	30 (19.1)	
1c	199 (53.8)	103 (48.4)	96 (61.1)	
2	124 (33.5)	100 (46.9)	24 (15.3)	
**Tumor focality**				**<0.001**^**2**^
Unifocal	292 (78.9)	154 (72.3)	138 (87.9)	
Multifocal	78 (21.1)	59 (27.7)	19 (12.1)	
**Histological subtype**				0.618^2^
NST	340 (91.9)	195 (91.5)	145 (92.4)	
ILC	11 (3.0)	6 (2.8)	5 (3.2)	
NST + ILC	10 (2.7)	5 (2.3)	5 (3.2)	
Other	9 (2.4)	7 (3.3)	2 (1.3)	
**ER status**				0.160^2^
ER+ (≥10 %)	274 (74.1)	151 (70.9)	123 (78.3)	
ER-	96 (25.9)	62 (28.2)	34 (21.7)	
**FDG-PET/CT**				**<0.001**^**2**^
Yes	183 (49.5)	178 (83.6)	5 (3.2)	
No	187 (50.5)	35 (16.4)	152 (96.8)	

ER, estrogen receptor; ILC, invasive lobular carcinoma; IQR, interquartile range; NAST, neoadjuvant systemic therapy; NST, no special type.

### 5.2 Upstaging by FDG-PET/CT scan

Of the 370 included patients, 183 patients (49.5 %) underwent an FDG-PET/CT prior to start of treatment and 187 (51.5 %) did not (Fig. 1). Patients receiving an FDG-PET/CT were younger (48y vs 52y), had a tumor ≥2 cm (49,2 % vs 18.2 %), and were more often treated with NAST followed by surgery instead of upfront surgery (97.3 % NAST and 2.7 % upfront surgery vs 13.4 % NAST and 86.6 % upfront surgery) **(Supplementary data,** Table 1**).**

In 7.7 % (14/183) of patients, FDG-PET/CT revealed regional lymph node involvement (n = 11) or distant metastases (n = 3). The majority of these patients (79 %, 11/14) had a tumor ≥2 cm. Seven patients had axillary and four patients had parasternal lymph node involvement, resulting in upstaging to cN1 and cN2b respectively. All 11 patients were treated in the neoadjuvant setting, predominantly receiving dual anti-HER2 blockade in combination with paclitaxel and carboplatin followed by targeted lymph node surgery (the MARI procedure) with or without additional RT [17]. In all 3 patients with distant metastases detected by FDG-PET/CT an oligo-metastatic treatment approach by means of radiotherapy (n = 2) or surgical removal (n = 1) of the metastatic lesion was applied.

### 5.3 Nodal involvement on final pathology after NAST or upfront surgery

From the 370 identified patients, 356 patients remained cN0M0 after ultrasound followed by FDG-PET/CT or ultrasound alone. Of these 356 patients, 55.9 % started with NAST (n = 199/356), while 44.1 % underwent upfront surgery (n = 157/356) according to physician's choice. Surgical treatment consisted mostly of breast-conserving surgery (BCS: 66 %) and a sentinel lymph node biopsy (SLNB: 99.2 %).

Overall, 42 out of 356 patients (11.8 %) were node-positive ((y)pN+) on final pathology: 6.0 % (12/199) had yppN + after NAST followed by surgery and 19.1 % (30/157) had pN + after upfront surgery (Fig. 1).

Among patients treated with NAST, 82 % (n = 164/199) underwent FDG-PET/CT prior to treatment. Nodal involvement on final pathology was observed in 6.1 % (n = 10/164, ypNmicro: 0.6 %, ypNmacro: 5.5 %) if FDG-PET/CT was performed and in 5.7 % (n = 2/35, all ypNmicro) if FDG-PET/CT was not performed.

On the contrary, among patients undergoing upfront surgery, the majority (97 %, n = 152/157) did not undergo FDG-PET/CT. Nodal involvement on final pathology was observed in 19.1 % (n = 29/152, pNmicro: 8.6 %, pNmacro: 10.5 %) if FDG-PET/CT was not performed and in 20 % (n = 1/5, all ypNmicro) if FDG-PET/CT was performed.

### 5.4 Nodal involvement on final pathology and breast pCR-rates after neoadjuvant PT

Among the 199 patients who started with NAST, 36.2 % (n = 72/199) patients received PT regimen. Other treatment regimens included predominantly PT combined with anthracyclines, or dual anti-HER2 blockade with paclitaxel and carboplatin (Supplementary data, Table 2).

Nodal involvement and breast pCR was evaluated separately for patients treated with neoadjuvant PT and for patients treated with other neoadjuvant regimens (Table 2). In patients treated with neoadjuvant PT, nodal involvement on final pathology was observed in 6.9 % of the patients (n = 5/72) whereas 5.5 % (n = 7/127) of patients treated with other regimens had nodal involvement (OR: 1.279, 95 % CI 0.391–4.188, p = 0.684)**.**

**Table 2:** Nodal involvement and pathological response in patients treated with neoajdvant systemic treatment. Either with paclitaxel + trastuzumab or other regimen. n = 199.

	NAST – all n = 199			NAST – PT n = 72			NAST – Other regimen n = 127		
	ypT0	ypT+	Total	ypT0	ypT+	Total	ypT0	ypT+	Total
**ypN0**	115	68	183	35	31	66	80	37	117
**ypN0 i+**	0	4	4	0	1	1	0	3	3
**ypN+**	0	12	12	0	5	5	0	7	7
**Total**	115	84	199	35	37	72	80	47	127
**ypT0**	115/199 (57.8 %)			35/72 (48.6 %)			80/127 (63.0 %)		
**ypN+**	12/199 (6.0 %)			5/72 (6.9 %)			7/127 (5.5 %)		

NAST, neoadjuvant systemic therapy; PT, paclitaxel + trastuzumab.

Overall, a pCR-rate of 57.8 % was observed; for patients treated with PT, pCR-rate was 48.6 % and among patients receiving other NAST regimens pCR-rate was 63.0 %, (Table 2, Supplementary data Table 2). Having a pCR was associated with more intense chemo regimens in univariable analysis, however in multivariable analyses this effect was no longer significant (univariable analysis OR 0.56 95 % CI 0.309–0.049, p = 0.049; multivariable analysis OR 0.82, 95 % CI 0.381–1.768, p = 0.614). Additionally, multivariable analyses revealed that having pCR was associated with having a grade 3 tumor and ER-tumor and a radiological complete response (Table 3).

**Table 3:** Univariable and multivariable logistic regression analysis on pathological complete response in patients treated with neoadjuvant systemic treatment. n = 199.

	Univariable analysis						Multivariable analysis			
	N of patients	N of events	OR	95 % CI		p-value	OR	95 % CI		P-value
Age										
<50	114	70	Ref				Ref			
≥50	85	45	0.707	0.400	1.249	0.232	0.740	0.349	1.565	0.430
**Grade**										
1–2	91	38	Ref				Ref			
3	102	72	3.347	1.845	6.074	<0.001	2.345	1.114	4.935	**0.025**
**Tumor size**										
T1	106	57	Ref							
T2	93	58	1.425	0.808	2.512	0.221				
**Tumor focality**										
Unifocal	147	86	Ref							
Multifocal	52	29	0.894	0.472	1.693	0.732				
**ER status**										
ER+	143	66	Ref				Ref			
ER-	56	49	8.167	3.465	19.251	<0.001	4.819	1.797	12.918	**0.002**
**Type of NST**										
Other	127	80	Ref				Ref			
PT	72	35	0.556	0.309	0.998	**0.049**	0.779	0.364	1.668	0.520
**rCR on MRI**										
No rCR	80	25	Ref				Ref			
rCR	103	80	7.652	3.946	14.838	<0.001	6.901	3.318	14.351	**<0.001**

ER, estrogen receptor; LN, lymph node; NST, neoadjuvant systemic treatment; pCR, pathological complete response; rCR; radiological complete response; US, ultrasound.

All patients with nodal involvement after NAST (6.0 %) had residual disease of the breast (non-pCR) after NAST.

### 5.5 Factors associated with nodal involvement on final pathology

Overall, the likelihood of having tumor-positive lymph nodes on final pathology was higher when undergoing upfront surgery (upfront surgery vs NAST), having a tumor of lower grade (grade 1–2 vs grade 3), and not performing FDG-PET/CT prior to start of treatment in univariable analyses**.** However, in the multivariable analysis only upfront surgery compared to NAST remained associated with increased risk of nodal involvement (OR 0.258, 95 % CI 0.069–0.972, p = 0.045) (Table 4). Type of neoadjuvant therapy (PT vs more intense regimen) was not associated with nodal involvement on.

**Table 4:** Univariable and multivariable logistic regression analysis on nodal involvement in all cN0 patients n = 356.

	Univariable analysis						Multivariable analyses			
	n of patients	n of events	OR	95 % CI		p-value	OR	95 % CI		p-value
Age										
<50	177	17	Ref				Ref			
≥50	179	25	1.528	0.794	1.528	0.204	1.380	0.696	2.738	0.357
**Grade**										
1–2	169	27	Ref				Ref			
3	181	15	0.475	0.243	0.928	**0.029**	0.515	0.253	1.051	0.068
**Tumor size**										
T1	239	31	Ref							
T2	117	11	0.696	0.337	1.440	0.329				
**Tumor focality**										
Unifocal	285	31	Ref							
Multifocal	71	11	1.502	0.714	3.158	0.283				
**Suspected LN on US**										
No	242	33	Ref							
Yes	112	9	0.553	0.255	1.200	0.134				
**ER status**										
ER+	266	36	Ref				Ref			
ER-	90	6	0.456	0.186	1.122	0.087	0.605	0.232	1.575	0.303
**NAST**										
No	157	30	Ref				Ref			
Yes	199	12	0.272	0.134	0.551	**<0.001**	0.258	0.069	0.972	**0.045**
**FDG-PET/CT**										
No	187	31	Ref				Ref			
Yes	169	11	0.350	0.170	0.722	**0.004**	1.197	0.307	4.671	0.795

LN, lymph node; NAST, neoadjuvant systemic treatment; US, ultrasound.

## 6 Discussion

In our study, we evaluated clinical upstaging by FDG-PET/CT and nodal involvement on final pathology in patients with small (≤3 cm), HER2+ BC who were node-negative on ultrasound. We observed clinical upstaging to cN + or cM+ in 7.7 % of patients after FDG-PET/CT, all of whom were cN0 on ultrasound. Additionally, the proportion of patients with nodal involvement on final pathology after FGD-PET/CT followed by NAST was 6.0 %, while nodal involvement after upfront surgery without FGD-PET/CT (which is the current standard of care according to international guidelines) was 20 %. Staging with FDG-PET/CT did not influence nodal involvement on final pathology. Nodal involvement and pCR-rates after neoadjuvant PT or a more intense neoadjuvant regimen were similar.

Current international guidelines from ESMO, NCCN, and ASCO recommend FDG-PET/CT in patients with breast cancer with nodal involvement and/or tumors larger than 5 cm [4,18,19]. Additionally, recently published EANM-SNMMI guidelines do not recommend FDG-PET/CT in stage I BC and recommend to consider FDG-PET/CT in stage IIA BC [15]. The rationale behind guidelines cautioning against routine FDG-PET/CT in early node-negative BC stems from retrospective analyses, which show high variation in sensitivity- and specificity rates in detecting metastasis by FDG-PET/CT compared to histopathology [[20], [21], [22]]. However, being a tertiary cancer center engaged in numerous trials within the neoadjuvant setting, we have integrated FDG-PET/CT into the diagnostic work-up fort all BC patients that are candidates for NAST. This deviation from standard practice raises the question whether this approach might be considered overtreatment or offers benefits to patients such as improved clinical staging prior to start of treatment.

In our study, nodal or distant metastases were found during clinical staging by FDG-PET/CT in 7.7 % of patients (n = 14) while being cN0 on ultrasound. The majority of these patients (79 %, 11/14) had a tumor ≥2 cm, considered stage IIA. Our findings are in line with recently published results by Francois et al. showing a change in treatment approach due to staging by FDG-PET/CT for 22 % of the patients with stage IIA (T2N0) HER2+BC, compared to only 10 % for the patients with stage I HER2+ BC [23]. The proportion of patients with nodal involvement after NAST with or without FDG-PET/CT were comparable (6.0 % and 5.7 % respectively), and no association between FDG-PET/CT and nodal involvement on final pathology was found in our multivariable analysis. However, only a small number (n = 35) of patients was treated with NAST without FDG-PET/CT. Although our observations suggest that the use of FDG-PET/CT in small, cN0 HER2+ breast cancer could result in upstaging in a subset of patients (7.7 %), its impact on nodal involvement after NAST remains uncertain.

The standard of care for patients with small, node-negative, HER2+ BC is upfront surgery followed by adjuvant PT, a regimen shown to be effective with a 7-year OS of 95 %.In contrast, for tumors larger than 2 cm or in case of nodal involvement, neoadjuvant systemic therapy is preferred over adjuvant therapy [5,24,25]. Up to 20 % of patients is upstaged to pN + on final pathology after upfront surgery and would not be offered adjuvant PT, These patients could have benefited from an extended chemotherapy with dual HER-blockade preferably in the neoadjuvant setting. A neoadjuvant treatment approach for small node-negative HER2+ BC may offer benefits such as early adaptation of the treatment regimen in case of progressive disease, breast- or axilla conserving surgery and response-guided adjuvant treatment. However, a limitation of the neoadjuvant approach is its reliance on accurate clinical staging to determine the extent of the neoadjuvant treatment schedule.

Since within our institute patients with small tumors are treated in the neoadjuvant setting, we were able to evaluate nodal involvement on final pathology in patients treated with NAST. Prior studies observed nodal involvement rates after NAST in HER2+ T1-2N0 breast cancer ranging from 10.25 % to 14.1 % [11,12]. Our slightly lower rates of nodal involvement could result from including patients with smaller tumors and upfront selection of patients with cN + after FDG-PET/CT prior to start of NAST. In contrast, nodal involvement on final pathology after upfront surgery was observed in 19.1 %, which is in line with findings from others where nodal involvement after upfront surgery in HER2+ T1-2N0 BC ranges from 19.8 % to 25.3 %. Taken together, these observations show that NAST contributes to eradication of occult axillary metastases, thereby reducing the rate of nodal involvement observed on final pathology. As node positive disease necessitates axillary radiotherapy or axillary lymph node dissection, there is a clear benefit for patients with small Her2+ breast cancer from NAST rather than upfront surgical treatment.

When considering a less intense neoadjuvant treatment approach with paclitaxel and trastuzumab (PT), we observed a pCR-rate of 48 %, which is slightly higher than previously reported pCR-rates by others, and nodal involvement on final pathology in only 7.1 % = of patients, suggesting that a neoadjuvant treatment approach with PT seems safe for patients with small tumors ≤3 cm. Hereby, we could potentially avoid intense treatment regimens [10].

Our study has some limitations; first, we made use of a retrospective cohort, which might lead to bias by indication resulting from the retrospective character of the cohort. Although it is local protocol to perform an FDG-PET/CT prior to NAST, some patients did not undergo FDG-PET/CT due to physician decision. Patients not undergoing FDG-PET/CT before NAST might have had more favorable characteristics. In addition, the exact reasons for upfront surgery or neoadjuvant chemotherapy are unknown and could therefore further affect our findings. Second, dissecting the individual effect of FDG-PET/CT and NAST on nodal status after this combined approach must be done with caution due to small sample sizes in particular for the group that received upfront surgery after FDG-PET/CT. Third, long-term follow-up data was not yet available for this cohort.

In summary, our hospital's preference to treat patients with small, node-negative, HER2-positve breast cancer in the neoadjuvant setting, combined with FDG-PET/CT, allowed us to evaluate nodal involvement in these patients. As the proportion of patients with positive lymph nodes after FDG-PET/CT and neoadjuvant PT was very small, this could be a safe treatment strategy for patients with small, node negative HER2+ BC. This strategy may result in less intense chemotherapy thereby reducing chemotherapy-induced toxicities, as well as reducing the need for extensive axillary treatment bij radiotheraoy or ALND. Additional research is warranted to evaluate whether this approach is preferable, as adjuvant PT, is still standard of care.

## CRediT authorship contribution statement

**Josefien P. van Olmen:** Writing – review & editing, Writing – original draft, Methodology, Investigation, Formal analysis, Data curation, Conceptualization. **Veerle CM. Geurts:** Writing – review & editing, Writing – original draft, Methodology, Investigation, Formal analysis, Data curation, Conceptualization. **Marie-Jeanne TFD. Vrancken Peeters:** Writing – review & editing, Methodology, Investigation, Conceptualization. **Caroline A. Drukker:** Writing – review & editing, Methodology, Investigation, Conceptualization. **Marcel PM. Stokkel:** Writing – review & editing. **Marleen Kok:** Writing – review & editing, Methodology, Investigation, Conceptualization. **Frederieke H. van Duijnhoven:** Writing – review & editing, Supervision, Methodology, Investigation, Conceptualization.

## 4 Ethics approval

This study was approved by the Institutional Review Board of the Netherlands Cancer Institute Antoni van Leeuwenhoek.All retrospective medical data studies at the Netherlands Cancer Institute have been executed pursuant to Dutch legislation and international standards. Prior to May 25, 2018, national legislation on data protection applied, as well as the International Guideline on Good Clinical Practice. From 25 May forward we also adhere to the GDPR. Within this framework, patients are informed and have always had the opportunity to object or actively consent to the (continued) use of their personal data & biospecimens in research. Hence, the procedures comply both with (inter-) national legislative and ethical standards.

## Funding

MK: Received research funding paid to the institute from: BMS, 10.13039/100004337Roche, 10.13039/100004325AstraZeneca. Advisory roles compensated to the institute for: AstraZeneca, Daiichi Sankyo, Domain Therapeutics, Alderaan, BMS, MSD, Roche outside the submitted work. Speakers’ fee compensated to the institute from: 10.13039/100004337Roche, BMS and 10.13039/100005564Gilead.

## Declaration of competing intrest

The authors declare that they have no known competing financial interests or personal relationships that could have appeared to influence the work reported in this paper.
