# Supplementary material for: Nodal involvement in patients with small, clinically node-negative HER2-positive breast cancer after staging with FDG-PET/CT and neoadjuvant systemic therapy
Source: Breast. 2024 Oct 18;78:103822. doi: 10.1016/j.breast.2024.103822 (PMC11539167; doi:10.1016/j.breast.2024.103822)
Supplement: Supplementary file 1 [file mmc1.docx]

**Supplemental data**

|  | **Total N = 370** | **PET-CT N = 183** | **No PET-CT** **N = 187** | **p-value** |
| --- | --- | --- | --- | --- |
| **Median age (IQR)** | 50 (41.75-59) | 48 (39-57) | 52 (43-61) | **0.013^1^** |
| **Tumor grade** |  |  |  | 0.532^2^ |
| 1 | 11 (3.0) | 6 (3.3) | 5 (2.7) |  |
| 2 | 163 (44.1) | 74 (40.4) | 89 (47.6) |  |
| 3 | 190 (51.4) | 100 (54.6) | 90 (48.1) |  |
| Missing | 6 (1.7) | 3 (1.6) | 3 (1.6) |  |
| **cT status** |  |  |  | **<0.001^2^** |
| 1a | 9 (2.4) | 2 (1.0) | 7 (3.7) |  |
| 1b | 38 (10.3) | 6 (3.3) | 32 (17.1) |  |
| 1c | 199 (53.8) | 85 (45.4) | 114 (61) |  |
| 2 | 124 (33.5) | 90 (49.2) | 34 (18.2) |  |
| **Tumor focality** |  |  |  | **<0.001^2^** |
| Unifcoal | 292 (78.9) | 129 (70.5) | 163 (87.2) |  |
| Multifocal | 78 (21.1) | 54 (29.5) | 24 (12.8) |  |
| **Histological subtype** |  |  |  | 0.967^2^ |
| NST | 340 (91.9) | 167 (91.3) | 173 (92.5) |  |
| ILC | 11 (3.0) | 6 (3.3) | 5 (2.7) |  |
| IDC+ILC | 10 (2.7) | 5 (2.7) | 5 (2.7) |  |
| Other | 9 (2.4) | 5 (2.7) | 4 (2.1) |  |
| **ER status** |  |  |  | 0.097^2^ |
| ER+ (≥10%) | 274 (74.1) | 128 (69.9) | 146 (78.1) |  |
| ER- | 96 (25.9) | 55 (30.1) | 41 (21.9) |  |
| **Management** |  |  |  | **<0.001^2^** |
| NAST | 213 (57.6) | 178 (97.3) | 35 (13.4) |  |
| Upfront surgery | 157 (42.4) | 5 (2.7) | 152 (86.6) |  |

**Table 1.** Utilization of FDG-PET/CT in all included patients. n = 370

*ER, estrogen receptor; ILC, invasive lobular carcinoma; IQR, interquartile range; NAST, neoadjuvant systemic therapy; NST, no special type; OR, odds ratio; PT, paclitaxel + trastuzumab*

| **NAST schedule** | **No. of patients (n = 199)** |
| --- | --- |
| Taxol + trastuzumab | 72 (36.2%) |
| Anthracyclines, Taxol + trastuzumab | 5 (2.5%) |
| PTC: Paclitaxel + carboplatin + trastuzumab | 16 (8%) |
| PTC-Ptz: Paclitaxel + carboplatin + trastuzumab + Pertuzumab | 83 (41.7%) |
| (F)EC followed by PTC-Ptz (Train II trial)  Fluoroucil, epirubicine, cyclophosphamide | 14 (7%) |
| Other  Cisplatin (1), switch form AC to PTC (2), taxol + (AC) herceptin + pertuzumab (3) Unknown (3) | 9 (4.5%) |

**Table 2.** Overview of neoadjuvant systemic treatment schedules. n =199

*AC, Adriamycin+Cyclophosphamide; NAST, neoadjuvant systemic treatment; PTC, paclitaxel+trastuzumab+carboplatin; Ptz, Pertuzumab*
